# Education, household income, and depressive symptoms in middle-aged and older Japanese adults

**DOI:** 10.1186/s12889-021-12168-8

**Published:** 2021-11-18

**Authors:** Aya Hinata, Keiko Kabasawa, Yumi Watanabe, Kaori Kitamura, Yumi Ito, Ribeka Takachi, Shoichiro Tsugane, Junta Tanaka, Ayako Sasaki, Ichiei Narita, Kazutoshi Nakamura

**Affiliations:** 1 Division of Preventive Medicine, Niigata University Graduate School of Medical and Dental Sciences, Niigata, Japan; 2 Department of Health Promotion Medicine, Niigata University Graduate School of Medical and Dental Sciences, Niigata, Japan; 3 Department of Food Science and Nutrition, Nara Women’s University Graduate School of Humanities and Sciences, Nara, Japan; 4 Center for Public Health Sciences, National Cancer Center, Tokyo, Japan; 5 Murakami Public Health Center, Niigata, Japan; 6 Division of Clinical Nephrology and Rheumatology, Niigata University Graduate School of Medical and Dental Sciences, Niigata, Japan

**Keywords:** Depression, Education, Income, Japan, Socioeconomic status

## Abstract

**Background:**

Income inequality has dramatically increased worldwide, and there is a need to re-evaluate the association between socio-economic status (SES) and depression. Relative contributions of household income and education to depression, as well as their interactions, have not been fully evaluated. This study aimed to examine the association between SES and depressive symptoms in Japanese adults, focusing on interactions between education and household income levels.

**Methods:**

This cross-sectional study used data from baseline surveys of two cohort studies. Participants were 38,499 community-dwelling people aged 40–74 years who participated in baseline surveys of the Murakami cohort study (2011–2012) and Uonuma cohort study (2012–2015) conducted in Niigata Prefecture, Japan. Information regarding marital status, education level, household income, occupation, activities of daily living (ADL), and history of cancer, myocardial infarction, stroke, and diabetes was obtained using a self-administered questionnaire. Depressive symptoms were examined using the Center for Epidemiologic Studies Depression Scale (CES-D). Logistic regression analysis was used to obtain odds ratios (ORs). Covariates included age, sex, marital status, education, household income, occupation, ADL, and disease history.

**Results:**

Individuals with higher education levels had lower ORs (adjusted P for trend = 0.0007) for depressive symptoms, independently of household income level. The OR of the university-or-higher group was significantly lower than that of the junior high school group (adjusted OR = 0.79). Individuals with lower household income levels had higher ORs (adjusted P for trend< 0.0001) for depressive symptoms, independently of education level. The type of occupation was not associated with depressive symptoms. In subgroup analyses according to household income level, individuals with higher education levels had significantly lower ORs in the lowest- and lower-income groups (adjusted P for trend = 0.0275 and 0.0123, respectively), but not in higher- and highest-income groups (0.5214 and 0.0915, respectively).

**Conclusions:**

Both education and household income levels are independently associated with the prevalence of depressive symptoms, with household income levels showing a more robust association with depressive symptoms than education levels. This suggests that a high household income level may offset the risk of depressive symptoms from having a low education level.

**Supplementary Information:**

The online version contains supplementary material available at 10.1186/s12889-021-12168-8.

## Background

Depression is a societal burden worldwide. The World Health Organization reported that more than 300 million people, or 4.4% of the global population, were living with depression in 2015, and that the prevalence of depression has increased by 18% in the last decade [1]. In Japan, a survey conducted by the Ministry of Health, Labour and Welfare found that the number of individuals suffering from mood disorders, including depression and bipolar disorder, was 1.3 million in 2017, which represents an increase of 38% from 2005 [2].

Depression and depressive symptoms in middle-aged and older adults play an important role in the course and outcomes associated with chronic health conditions [3]. Several studies have found the aging experience to be a key contributor to depression in middle-aged and older populations [4–6]. Social factors impact the aging experience, and influence the vulnerability to disorders such as psychiatric morbidity [7, 8]. Notably, socio-economic status (SES) is a well-documented risk factor for depression and depressive symptoms [9–15]. However, findings appear to vary across studies.

Income inequality has dramatically increased worldwide in the last decades [16], and consequently, there is a need to re-evaluate the association between SES and depression/depressive symptoms. Income inequality is related to the feeling of unfairness, stress, and anxiety [17, 18], and could lead to psychological problems including depression. Indeed, the significant increase in income inequity in Japan over the last few decades might have affected the association between income and depression [19].

Three dimensions of SES, namely, income, education, and occupation, are associated with depression/depressive symptoms. However, the strength of the associations differ by region or culture [9–11]. Some European studies have found education level to be a more important factor related to depressive symptoms than household income or occupation among older adults [12, 13]. Another study identified both education level and household income, but not occupation, as important predictors of depressive symptoms in adults aged 35–74 years [14]. For Asian populations aged ≥65 years, a study conducted in Korea demonstrated that low education levels and manual occupation were associated with depression [15], and a study conducted two decades ago in Japan reported that income, but not education, was associated with depressive symptoms [20]. Meanwhile, a study in China reported that low education and income levels were associated with depression/depressive symptoms in older adults (aged ≥60 years) [21, 22]. Recently, type of occupation was identified as a potential factor associated with depressive symptoms [23, 24]. However, the relative contributions of income, education, and occupation to depression/depressive symptoms are unclear, and there exists a research gap that warrants further investigation.

Against this backdrop, the present study aimed to examine the association of education and household income levels with depression in two large Japanese population-based cohorts, the Murakami and Uonuma cohorts, from Niigata, Japan. The results of this study may help fill the research gap on the association between SES and depression.

## Methods

### Study design and participants

The present study was cross-sectional in design. Participants were selected from among community-dwelling people aged 40–74 years who participated in the baseline surveys of the Murakami cohort study [25] and Uonuma cohort study [26] conducted in Niigata Prefecture, Japan. The Murakami cohort study originally targeted participants aged 40–74 years at baseline. Accordingly, we used the same age range, although participants of the Uonuma cohort study were those aged 40 years and older. All of the 34,802 and 49,261 community residents living in the Murakami and Uonuma areas, respectively, were invited to participate in this study, and 14,364 (41.2%) and 33,264 (67.5%) participated in the respective baseline surveys. Among the total of 47,628 participants, 38,499 (11,490 for Murakami and 27,009 for Uonuma) who did not have missing data were analysed as participants of the present study. All participants provided written informed consent. The protocol of this study was approved by the Ethics Committee of Niigata University (No. 2018–0379).

### Procedure

The baseline survey was conducted in 2011–2012 in the Murakami area and 2012–2015 in the Uonuma area. Details of these baseline surveys have been published elsewhere [25, 26]. Briefly, a self-administered questionnaire in paper format was distributed to participants and collected through a community-based communication network or postal mail. The questionnaire requested information regarding marital status, education level, household income, occupation, activities of daily living (ADL), and history of cancer, myocardial infarction, stroke, and diabetes. Marital status was categorized as 1) married, 2) never married, and 3) divorced, separated, or bereaved. Education level was categorized as 1) junior high school, 2) high school, 3) junior/vocational college, and 4) university or higher. Household income (yen) per year was categorized as 1) 0–2,999,000 (lowest group), 2) 3,000,000-5,999,000 (2nd group), 3) 6,000,000-8,999,000 (3rd group), and 4) ≥9,000,000 (highest group) (USD $1 roughly equivalent to 110 yen in 2020). ADL was categorized as 1) no disability, 2) some disability but able to live and go out independently, or live inside almost independently but able to go out with assistance, and 3) able to live inside with assistance and stay in bed almost all day, or lying in bed all day with assistance for toilet use, feeding, and dressing. Depressive symptoms were assessed using the Center for Epidemiologic Studies Depression Scale (CES-D) [27], a short self-report scale designed to measure depressive symptomatology during the previous week in the general population. This study used the short form of CES-D (11-items in 3 categories [0, 1, 2]), with scores ≤6 corresponding to lower levels of depression and scores ≥7 corresponding to higher levels of depression [28, 29]. The validity of the 11-item CES-D was previously tested by comparing factor loadings between the standard 20-item CES-D and the 11-item CES-D [29]. In the present study, the standardized Cronbach’s alpha value for the 11-item CES-D was 0.82, which is acceptably high.

### Statistical methods

Data are presented as mean ± standard deviation (SD) for continuous variables. Simple and multiple logistic regression analyses were used to obtain unadjusted and adjusted odds ratios (ORs), respectively, for having depressive symptoms. In the multivariate analysis, covariates included age (continuous variable), sex, marital status (dummy variable), education level, household income, occupation (dummy variable), and history of each disease. Spearman’s correlation coefficient between education and income was 0.241 (*P* < 0.0001). Interactions between education and income were evaluated by multiple logistic regression analysis, by adding the interaction term to the above-mentioned covariates. P-for-trend values were calculated using logistic regression analysis. SAS statistical software (release 9.4, SAS Institute Inc., Cary, NC, USA) was used for statistical analyses. *P* < 0.05 was considered statistically significant.

## Results

Depressive symptoms were observed in 31.8% of participants (mean age: 57.7 years, 49.3% women). The prevalence of depressive symptoms by age group was 38.7% among participants in their 40’s, 35.9% among those in their 50’s, 26.3% among those in their 60’s, and 24.0% among those in their 70’s. Table 1 shows the distribution of participant characteristics according to education and income levels. Among participants with the highest education level (university or higher), more participants were male, were married, had a higher household income, and were engaged in office work or professional work, and fewer participants were physically dependent, compared to participants in the other three categories. Among participants with the highest household income level (9,000,000 yen/year), more participants were married, had higher education levels, were engaged in office work or professional work, and had no disability, compared to participants in the other three categories. The characteristics of all participants are shown in Additional Table 1.

**Table 1:** Distribution of participant characteristics (number with percent) according to education and income levels

	Education level					Household income (Yen/yr)				
	Junior high School	High school	Junior college	University or higher	P for trend	<3,000,000	≥3,000,000; < 6,000,000	≥6,000,000; < 9,000,000	≥9,000,000	P for trend
	*N* = 9478	*N* = 19,098	*N* = 6859	*N* = 3064		*N* = 12,986	*N* = 15,975	*N* = 6265	*N* = 3273	
Male sex	4858 (51.3%)	9881 (51.7%)	2454 (35.8%)	2311 (75.4%)	< 0.0001	5913 (45.5%)	8659 (54.2%)	3274 (52.3%)	1658 (50.7%)	< 0.0001
Age (years)										
< 50	476 (5.0%)	4919 (25.8%)	2345 (34.2%)	904 (29.5%)	< 0.0001	2116 (16.3%)	4122 (25.8%)	1712 (27.3%)	694 (21.2%)	< 0.0001
50–59	1530 (16.1%)	6537 (34.2%)	2634 (38.4%)	1104 (36.0%)		3049 (23.5%)	4801 (30.1%)	2554 (40.8%)	1401 (42.8%)	
60–69	5108 (53.9%)	6242 (32.7%)	1612 (23.5%)	843 (27.5%)		5749 (44.3%)	5491 (34.4%)	1616 (25.8%)	949 (29.0%)	
≥ 70	2364 (24.9%)	1400 (7.3%)	268 (3.9%)	213 (7.0%)		2072 (16.0%)	1561 (9.8%)	383 (6.1%)	229 (7.0%)	
Marital status										
Married	7486 (79.8%)	15,517 (81.6%)	5530 (80.9%)	2532 (83.0%)	< 0.0001^2^	8877 (69.0%)	13,386 (84.2%)	5701 (91.3%)	3101 (94.9%)	< 0.0001^2^
Never married	552 (5.9%)	1518 (8.0%)	569 (8.3%)	310 (10.2%)		1597 (12.4%)	1084 (6.8%)	223 (3.6%)	45 (1.4%)	
Divorced, separated, or bereaved	1348 (14.4%)	1976 (10.4%)	736 (10.8%)	209 (6.9%)		2399 (18.6%)	1428 (9.0%)	321 (5.1%)	121 (3.7%)	
Education level										
Junior high school	–	–	–	–		4879 (37.6%)	3353 (21.0%)	825 (13.2%)	421 (12.9%)	< 0.0001
High school	–	–	–	–		5807 (44.7%)	8468 (53.0%)	3259 (52.0%)	1564 (47.8%)	
Junior college	–	–	–	–		1777 (13.7%)	2990 (18.7%)	1364 (21.8%)	728 (22.2%)	
University or higher	–	–	–	–		523 (4.0%)	1164 (7.3%)	817 (13.0%)	560 (17.1%)	
Household income (Yen/yr)										
0–2,990,000	4879 (51.5%)	5807 (30.4%)	1777 (25.9%)	523 (17.1%)	< 0.0001	–	–	–	–	
3,000,000-5,990,000	3353 (35.4%)	8468 (44.3%)	2990 (43.6%)	1164 (38.0%)		–	–	–	–	
6,000,000-8,990,000	825 (8.7%)	3259 (17.1%)	1364 (19.9%)	817 (26.7%)		–	–	–	–	
≥ 9,000,000	421 (4.4%)	1564 (8.2%)	728 (10.6%)	560 (18.3%)		–	–	–	–	
Occupation^1^										
Sales and service	1331 (14.2%)	3683 (19.4%)	1413 (20.7%)	439 (14.4%)	< 0.0001^3^	2404 (18.7%)	3079 (19.4%)	930 (15.0%)	453 (13.9%)	< 0.0001^3^
Office work	117 (1.3%)	1957 (10.3%)	691 (10.1%)	368 (12.1%)		617 (4.8%)	1203 (7.6%)	845 (13.6%)	468 (14.4%)	
Professional	1224 (13.0%)	3616 (19.1%)	2550 (37.4%)	1310 (42.9%)		1476 (11.5%)	3865 (24.4%)	2098 (33.7%)	1261 (38.8%)	
Manual	2648 (28.2%)	4863 (25.6%)	705 (10.3%)	245 (8.0%)		3020 (23.4%)	3679 (23.2%)	1187 (19.1%)	575 (17.7%)	
No job or others	4066 (43.3%)	4853 (25.6%)	1463 (21.5%)	689 (22.6%)		5374 (41.7%)	4040 (25.5%)	1161 (18.7%)	496 (15.3%)	
Area										
Murakami	2791 (29.5%)	6195 (32.4%)	1754 (25.6%)	750 (24.5%)	< 0.0001	4078 (31.4%)	4650 (29.1%)	1851 (29.6%)	911 (27.8%)	< 0.0001
Uonuma	6687 (70.6%)	12,903 (67.6%)	5105 (74.4%)	2314 (75.5%)		8908 (68.6%)	11,325 (70.9%)	4414 (70.5%)	2362 (72.2%)	
Disease history of										
Cancer	770 (8.1%)	1144 (6.0%)	384 (5.6%)	185 (6.0%)	< 0.0001	941 (7.3%)	975 (6.1%)	368 (5.9%)	199 (6.1%)	0.0002
Myocardial infarction	80 (0.8%)	107 (0.6%)	23 (0.3%)	20 (0.7%)	0.0035	91 (0.7%)	93 (0.6%)	27 (0.4%)	19 (0.6%)	0.0722
Stroke	266 (2.8%)	352 (1.8%)	91 (1.3%)	58 (1.9%)	< 0.0001	365 (2.8%)	273 (1.7%)	88 (1.4%)	41 (1.3%)	< 0.0001
Diabetes	955 (10.1%)	1238 (6.5%)	334 (4.9%)	213 (7.0%)	< 0.0001	1085 (8.4%)	1058 (6.6%)	392 (6.3%)	205 (6.3%)	< 0.0001
Activities of daily living										
No disability	8595 (90.7%)	18,215 (95.4%)	6582 (96.0%)	2925(95.5%)	< 0.0001	11,817 (91.0%)	15,221 (95.3%)	6092 (97.2%)	3187(97.4%)	< 0.0001
Having some disability^4^	809 (8.5%)	823 (4.3%)	261 (3.8%)	129 (4.2%)		1079 (8.3%)	701 (4.4%)	162 (2.6%)	80 (2.4%)	
Physically dependent^5^	74 (0.8%)	60 (0.3%)	16 (0.2%)	10 (0.3%)		90 (0.7%)	53 (0.3%)	11 (0.2%)	6 (0.2%)	

^1^Professional includes professional and management; manual includes security, farming/forestry/fishery, transportation, and labour services

^2^Trend of married persons

^3^Trend of manual workers

^4^Having some disability, but can go out independently

^5^Having disability, and cannot go out without assistance

Prevalence and ORs for having depressive symptoms according to education level by sex are shown in Table 2. Participants with higher education levels had lower final-adjusted ORs (P for trend = 0.0494 for men, P for trend = 0.0148 for women, P for trend = 0.0007 for men and women combined). Adjusted ORs of the university-or-higher group were significantly lower than those of the junior high school group (reference) in men (final-adjusted OR = 0.80, 95%CI: 0.71–0.90), women (final-adjusted OR = 0.79, 95%CI: 0.66–0.95), and men and women combined (final-adjusted OR = 0.79, 95%CI: 0.72–0.87).

**Table 2:** Prevalence and odds ratios (ORs) for depressive symptoms according to education level, adjusted for income and other potential confounders

	Education level				P for trend
	Junior high school	High school	Junior college	University or higher	
Men					
Prevalence	1385/4858 (28.5%)	2917/9881(29.5%)	886/2454 (36.1%)	612/2311(26.5%)	
Age-adjusted OR (95% CI)	1 (Reference)	0.83 (0.77–0.90)	1.02 (0.91–1.14)	0.68 (0.61–0.77)	< 0.0001
Adjusted OR (1)^1^ (95% CI)	1 (Reference)	0.88 (0.81–0.95)	1.06 (0.94–1.18)	0.73 (0.65–0.82)	0.0005
Adjusted OR (2)^2^ (95% CI)	1 (Reference)	0.92 (0.85–1.00)	1.10 (0.98–1.24)	0.80 (0.71–0.91)	0.0494
Women					
Prevalence	1478/4620 (32.0%)	3198/9217 (34.7%)	1518/4405 (34.5%)	229/753 (30.4%)	
Age-adjusted OR (95% CI)	1 (Reference)	0.89 (0.82–0.96)	0.83 (0.75–0.92)	0.69 (0.58–0.82)	< 0.0001
Adjusted OR (1)^1^ (95% CI)	1 (Reference)	0.93 (0.85–1.01)	0.87 (0.79–0.96)	0.72 (0.60–0.86)	0.0002
Adjusted OR (2)^2^ (95% CI)	1 (Reference)	0.96 (0.88–1.05)	0.92 (0.83–1.02)	0.79 (0.66–0.95)	0.0148
Men and women combined					
Prevalence	2863/9478 (30.2%)	6115/19098 (32.0%)	2404/6859 (35.1%)	841/3064 (27.5%)	
Age-adjusted OR (95% CI)	1 (Reference)	0.85 (0.81–0.90)	0.90 (0.84–0.97)	0.66 (0.60–0.72)	< 0.0001
Adjusted OR (1)^3^ (95% CI)	1 (Reference)	0.90 (0.85–0.96)	0.93 (0.86–1.00)	0.72 (0.65–0.79)	< 0.0001
Adjusted OR (2)^4^ (95% CI)	1 (Reference)	0.94 (0.89–1.00)	0.97 (0.90–1.05)	0.79 (0.72–0.87)	0.0007

^1^Adjusted for age, marital status, occupation, area, disease history, and ADL

^2^Adjusted for age, marital status, occupation, area, disease history, ADL, and income

^3^Adjusted for sex, age, marital status, occupation, area, disease history, and ADL

^4^Adjusted for sex, age, marital status, occupation, area, disease history, ADL, and income

Prevalence and ORs for depressive symptoms according to income level by sex are shown in Table 3. Participants with lower income levels had higher adjusted ORs (P for trend< 0.0001 for men, women, and men and women combined), and all adjusted ORs were significantly lower than that of the lowest income group (reference; final-adjusted OR = 0.59, 95%CI: 0.51–0.68 for men, final-adjusted OR = 0.68, 95%CI: 0.60–0.77 for women, final-adjusted OR = 0.64, 95%CI: 0.59–0.70 for men and women combined).

**Table 3:** Prevalence and odds ratios (ORs) for depressive symptoms according to income level, adjusted for education and other potential confounders

	Household income (Yen/yr)				P for trend
	Lowest	2nd	3rd	Highest	
	(<3,000,000)	(≥3,000,000; < 6,000,000)	(≥6,000,000; < 9,000,000)	(≥9,000,000)	
Men					
Prevalence	2043/5913 (34.6%)	2532/8659 (29.2%)	854/3274 (26.1%)	371/1658 (22.4%)	
Age-adjusted OR (95% CI)	1 (Reference)	0.66 (0.61–0.71)	0.53 (0.48–0.59)	0.46 (0.40–0.52)	< 0.0001
Multivariable adjusted OR (1)^1^ (95% CI)	1 (Reference)	0.76 (0.71–0.83)	0.66 (0.59–0.74)	0.58 (0.50–0.66)	< 0.0001
Multivariable adjusted OR (2)^2^ (95% CI)	1 (Reference)	0.77 (0.71–0.83)	0.67 (0.60–0.75)	0.59 (0.51–0.68)	< 0.0001
Women					
Prevalence	2649/7073 (37.5%)	2437/7316 (33.3%)	878/2991 (29.4%)	459/1615 (28.4%)	
Age-adjusted OR (95% CI)	1 (Reference)	0.78 (0.73–0.84)	0.62 (0.56–0.68)	0.61 (0.54–0.68)	< 0.0001
Multivariable adjusted OR (1)^1^ (95% CI)	1 (Reference)	0.83 (0.77–0.89)	0.67 (0.61–0.74)	0.67 (0.59–0.76)	< 0.0001
Multivariable adjusted OR (2)^2^ (95% CI)	1 (Reference)	0.84 (0.78–0.90)	0.68 (0.62–0.75)	0.68 (0.60–0.77)	< 0.0001
Men and women combined					
Prevalence	4692/12986 (36.1%)	4969/15975 (31.1%)	1732/6265 (27.7%)	830/3273 (25.4%)	
Age-adjusted OR (95% CI)	1 (Reference)	0.72 (0.68–0.75)	0.57 (0.54–0.61)	0.53 (0.49–0.58)	< 0.0001
Multivariable adjusted OR (1)^3^ (95% CI)	1 (Reference)	0.80 (0.76–0.85)	0.67 (0.63–0.72)	0.63 (0.57–0.69)	< 0.0001
Multivariable adjusted OR (2)^4^(95% CI)	1 (Reference)	0.81 (0.77–0.85)	0.68 (0.64–0.73)	0.64 (0.59–0.70)	< 0.0001

3,000,000 yen is roughly equivalent to 28,000 US dollars (in 2020)

^1^Adjusted for age, marital status, occupation, area, disease history, and ADL

^2^Adjusted for age, marital status, occupation, area, disease history, ADL, and education

^3^Adjusted for sex, age, marital status, occupation, area, disease history, and ADL

^4^Adjusted for sex, age, marital status, occupation, area, disease history, ADL, and education

Prevalence and ORs for depressive symptoms according to education level stratified by household income in men and women are shown in Table 4. Participants with higher education levels had lower ORs in the lowest- (P for trend = 0.0275) and 2nd-income (P for trend = 0.0123) groups, but not in the 3rd- (P for trend = 0.5214) and highest-income (P for trend = 0.0915) groups. This suggests a potential interaction, albeit non-significant, between education and income (adjusted *P* = 0.2851).

**Table 4:** Prevalence and odds ratios (ORs) for depressive symptoms according to education level, stratified by household income in men and women

	Education level				P for trend
Household income	Junior high school	High school	Junior college	University or higher	
Lowest (<3,000,000 Yen/yr)					
Prevalence	1666/4879 (34.2%)	2146/5807 (40.0%)	693/1777 (39.0%)	187/523 (35.8%)	
Adjusted OR^1^ (95% CI)	1 (Reference)	0.92 (0.84–1.00)	0.90 (0.79–1.02)	0.86 (0.70–1.04)	0.0275
2nd (≥3,000,000, < 6,000,000 Yen/yr)					
Prevalence	930/3353 (27.7%)	2672/8468 (31.6%)	1047/2990 (35.0%)	320/1164 (27.5%)	
Adjusted OR^1^ (95% CI)	1 (Reference)	0.94 (0.86–1.04)	1.00 (0.89–1.12)	0.74 (0.63–0.86)	0.0123
3rd (≥6,000,000, < 9,000,000 Yen/yr)					
Prevalence	174/825 (21.1%)	905/3259 (27.8%)	444/1364 (32.6%)	209/817 (25.6%)	
Adjusted OR^1^ (95% CI)	1 (Reference)	1.12 (0.92–1.37)	1.28 (1.02–1.60)	0.89 (0.70–1.15)	0.5214
Highest (≥9,000,000 Yen/yr)					
Prevalence	93/421 (22.1%)	392/1564 (25.1%)	220/728 (30.2%)	125/560 (22.3%)	
Adjusted OR^1^ (95% CI)	1 (Reference)	0.95 (0.72–1.25)	1.10 (0.81–1.51)	0.71 (0.51–1.00)	0.0915

3,000,000 yen was roughly equivalent to 28,000 US dollars in 2020

^1^Adjusted for sex, age, marital status, occupation, area, disease history, and ADL

There was no significant association between type of occupation and depressive symptoms. Final multivariable-adjusted ORs and 95%CIs for depressive symptoms were 1.06 (0.97–1.17) for office work, 1.06 (0.99–1.14) for professional work, 1.04 (0.96–1.11) for manual work, and 0.99 (0.93–1.07) for no job or others, relative to sales and service (reference).

**Fig. 1:**
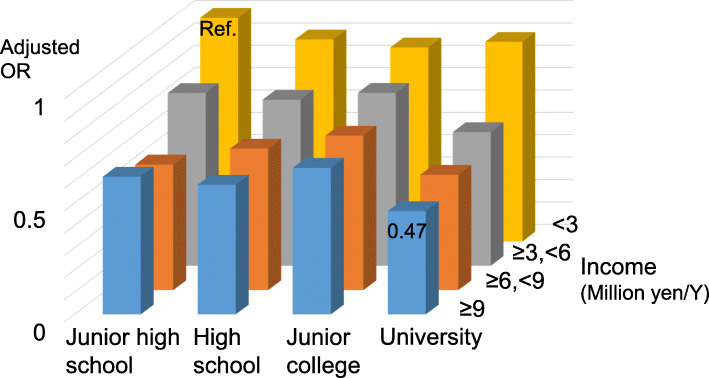
Odds ratios (ORs) according to education and income levels. ORs were adjusted for age, sex, marital status, occupation, area, ADL, and history of cancer, myocardial infarction, stroke, and diabetes. The association of income level with depressive symptoms was more robust than that of education level

Finally, adjusted ORs according to both education and income levels are shown in Fig. 1. Individuals with a “university” education in the “≥9 million yen” group had the lowest OR (0.47, 95%CI: 0.38–0.58) relative to those with a “junior high school” education in the “<3 million yen” group (reference). ORs of the other levels were significantly lower than the reference, except for those with a “university” education in the “<3 million yen” group.

## Discussion

The main findings of the present study were as follows: 1) high education levels were associated with a low prevalence of depressive symptoms independently of income level, 2) high income levels were more robustly associated with a low prevalence of depressive symptoms than education level, and 3) an association between education level and prevalence of depressive symptoms was observed in low income groups, but not in high income groups, suggesting a potential interaction.

A meta-analysis of 24 cross-sectional studies published in 2010 [30] reported that high education levels are significantly associated with a decreased risk of depression, and results of the present study are consistent with this. However, the association observed in the present study was weaker (adjusted OR = 0.79 for highest vs. lowest education group) than that reported in the meta-analysis (OR = 0.63 [95%CI: 0.55–0.72]) for depression in those with high education levels than those with low education levels. This weaker association may be a characteristic of the Japanese population, as further evidenced by an epidemiologic study reporting a null association between education level and depression [20]. In the present study, high education levels were associated with a lower prevalence of depressive symptoms independently of income level. However, only a few epidemiologic studies have assessed whether education and income levels are independently associated with depressive symptoms in population-based samples. Schlax et al. [14] reported that education level was associated with elevated depressive symptoms independently of income level in 12,484 Germans aged 35–74 years. Similarly, Domènech-Abella et al. [13] showed that education level was associated with depression independently of income level, and suggested that the association between education and depression was mediated by behavioural factors. Higher education is considered to provide a problem-solving attitude about health and access to medical or preventive health services [31]. Moreover, education can be considered a marker of childhood SES, as it has been shown to improve health literacy and the ability to master stressful and demanding situations in adulthood [32]. Contrary to these reports from socioeconomically advantaged countries, Cermakova et al. [33] demonstrated that education in the Czech Republic, a non-socioeconomically advantaged country, was not an independent, correlating factor of depressive symptoms in 6964 Czechs aged 45–69 years. This suggests that the independent effects of education and income on depressive symptoms may specifically be observed in socioeconomically advantaged countries.

There is a large body of evidence linking income levels with depression. A meta-analysis published in 2003 [9] showed that high income levels are significantly associated with a decreased risk of depression, with crude ORs of 0.55–0.60 (highest vs. lowest). The present findings on the association between income level and depression are in line with those of the previous meta-analysis. Comparable data from a group in Korea were published in 2018 [34], with an adjusted OR for depressive symptoms of 0.54 (highest vs. lowest quartile). In contrast, only a few epidemiologic studies on this topic have been conducted in Japan. In the present study, household income level was associated with depressive symptoms in both men and women independently of education level. A previous Japanese study conducted in people aged ≥65 years in 2003 reported that the OR for depression, diagnosed by the Geriatric Depression Scale (GDS-15), of high income (≥4 million yen/year) relative to low income (< 2 million yen/year) was 0.43 [20].

Although the present study found that education and income levels were independently associated with depressive symptoms, ORs of education levels for depressive symptoms were attenuated toward null after adjusting for household income (Table 2), likely due to an intercorrelation between education and income levels (Spearman’s correlation coefficient = 0.241). The association of household income with depressive symptoms was more robust than that of education level, as reflected by the negligible change in ORs of household income after adjusting for education level (Table 3). The robustness of the association between household income (rather than education level) and depressive symptoms is clearly shown in Fig. 1. In contrast, a recent European study [13] reported that education level is more strongly correlated with depression than household income. This discrepancy may be due to the fact that the European study [13] included a “no formal education” group in the education category, which had a major impact on depression that was not observed in the present study. Another possible explanation is that a regional or cultural difference exists. Indeed, other East Asian studies have shown that income level was associated with depressive symptoms, while education level was not [20, 35].

Education level was not significantly associated with depressive symptoms in the highest-income group (Table 4). Education is thought to shape psychosocial resources, such as social support and self-efficacy, which reduce stress and develop health-promoting behaviours, while income provides a resource for coping with ill health by enabling a person to access health care [36]. The findings of the present study may suggest that a high household income level can offset the risk of depressive symptoms in those with a low education level.

With regard to the mechanism underlying the association between SES and depression, a low SES may cause depressive symptoms, for example, via high levels of perceived stress and exposure to poor environmental factors [37], which can lead to reduced serotonergic function and subsequent depression [38]. Epigenetic modification of gene expression associated with low SES has also been reported to predict changes in depression-related brain function [39].

There may have been selection bias in the present study, given that the participation rate was not high. The prevalence of CES-D-based depressive symptoms was higher in participants in their 40’s (38.7%) and 50’s (35.9%) relative to those reported for a representative Japanese population (estimated response rates of about 87–89%; 27.9% among people in their 40’s, 26.6% among those in their 50’s) [40]. However, participants in their 60’s and 70’s showed a similar or lower prevalence of depressive symptoms (26.3 and 24.0%, respectively) compared to the representative Japanese population (60’s: 24.8%, 70’s: 36.0%) [40]. A potential explanation is that participation rates among those in their 40’s and 50’s were considerably lower compared to those in their 60’s and 70’s [26]. In sum, the prevalence of depressive symptoms among the middle-aged participants of the present study may have been overestimated, which could have affected the ORs.

The overall prevalence of CES-D-based depressive symptoms was high (31.8%) in the present study. A previous study using the CES-D [40] also reported a higher prevalence (28.1%) of depressive symptoms in a representative Japanese adult population compared to Western countries (≤ 20%). This may be explained by a tendency of Japanese people to suppress the expression of positive affect [41], suggesting an overestimation of depressive symptoms.

The present study has some strengths. The large sample size from two independent population-based cohorts enabled us to conduct an adequately powered and statistically robust study, even for subgroup analyses. In addition, the prevalence of depression has been reported to peak in older adulthood (above 7.5% among females and 5.5% among males aged 55–74 years) [1]. Therefore, the findings of the present study, which targeted people aged 40–74 years, have a potential public health impact.

However, this study also has some limitations worth noting. First, the regions from which the two cohorts were sampled have medium- and small-sized local governments, and thus, the results of the present study may not apply to regions with large or metropolitan governments (e.g., Tokyo), where SES of residents differs from that of the present cohorts. Second, selection bias other than the low participation rate may have influenced the results of statistical analyses. People with lower education and/or income levels may have participated less than those with higher levels, and individuals suffering from serious depressive symptoms are less likely to participate in epidemiologic studies. Third, comorbidities such as dementia may have influenced the results. Although the present study did not examine the history of dementia of participants, only 3 (0.03%) suspected dementia cases were identified in the Murakami cohort (from a long-term care insurance database). Therefore, the impact of cognitive impairment on the results of the present study is likely minimal. Fourth, this study was based on self-reported information from participants. Thus, misclassification may have occurred, and the strength of the association between exposure and outcome may be attenuated toward null. Fifth, demographic and lifestyle factors were statistically adjusted for, but other potential confounders were not considered. For example, interpersonal conflicts [42], personality [43], and social isolation [44] have recently been reported to be influential factors. Further studies will be needed to address these factors. Finally, causal relationships could not be determined due to the cross-sectional design. Longitudinal studies to confirm the results of the present study are warranted.

## Conclusion

Both education and household income levels were independently associated with the prevalence of depressive symptoms, and income level was more robustly associated with depressive symptoms than education level. A high household income level might offset the risk of depressive symptoms in those with a low education level. These findings may contribute to efforts toward the primary prevention of depressive symptoms.

The present study has the following implications. First, the findings further support the robust association of SES, particularly education and household income, with depressive symptoms. Although no significant interaction was observed, participants with the lowest and second lowest household income levels were more likely to be subject to the effect of education level on depression. This could provide a potential target population to develop additional strategies for the prevention of depression in community-dwelling middle-aged and older adults.

## Supplementary Information

**Additional file 1.**

## Data Availability

The datasets generated and/or analysed during the current study are not publicly available because participants did not consent to have their data provided to anyone outside the research group. However, the minimal dataset may be available upon ethical approval by the Ethics Committee of Niigata University. Please contact the Office of the Murakami cohort study (kazun@med.niigata-u.ac.jp) or the Uonmua cohort study (yumii@med.niigata-u.ac.jp) for inquiries about data availability.

## Abbreviations

CES-D: Center for Epidemiologic Studies Depression Scale
SES: Socioeconomic status
GDS: Geriatric Depression Scale
OR: Odds ratio
SAS: Statistical Analysis System
SD: Standard deviation
USA: United States of America

## References

[1] WHO. Depression and Other Common Mental Disorders. Geneva: WHO; 2017.

[2] Health, Labour and Welfarre Statistics Association Kokumin-Eisei-no-Doko. 2019/2020. Tokyo: Health, Labour and Welfarre Statistics Association; 2019.

[3] Chapman DP, Perry GS, Strine TW (2005). The vital link between chronic disease and depressive disorders. Prev Chronic Dis.

[4] Prince MJ, Harwood RH, Blizard RA, Thomas A, Mann AH (1997). Social support deficits, loneliness and life events as risk factors for depression in old age. The gospel oak project VI. Psychol Med.

[5] Wei QQ, Qian S, Yu LJ, Xia JR, Quan WY, Xu Y (2020). Risk factors for depressive symptoms among older Chinese adults: a meta-analysis. J Affect Disord.

[6] Abuladze L, Opikova G, Lang K (2020). Factors associated with incidence of depressiveness among the middle-aged and older Estonian population. SAGE Open Med.

[7] Baru A, Murugan P (2016). Social determinants of vulnerability to ill-health: evidences from Mendi town, Western Ethiopia. J Health Soc Sci.

[8] Mursal A, Dong W (2018). Education as a key determinant of health: a case study from rural Anhui, China. J Health Soc Sci.

[9] Lorant V, Deliège D, Eaton W, Robert A, Philippot P, Ansseau M (2003). Socioeconomic inequalities in depression: a meta-analysis. Am J Epidemiol.

[10] Ogundare T (2019). Culture and mental health: towards cultural competent mental health delivery. J Health Soc Sci..

[11] Chirico F, Heponiemi T, Pavlova M, Zaffina S, Magnavita N (2019). Psychosocial risk prevention in a global occupational health perspective. A descriptive analysis. Int J Environ Res Public Health.

[12] Chlapecka A, Kagstrom A, Cermakova P (2020). Educational attainment inequalities in depressive symptoms in more than 100,000 individuals in Europe. Eur Psychiatry.

[13] Domènech-Abella J, Mundó J, Leonardi M, Chatterji S, Tobiasz-Adamczyk B, Koskinen S, Ayuso-Mateos JL, Haro JM (2018). The association between socioeconomic status and depression among older adults in Finland, Poland and Spain: a comparative cross-sectional study of distinct measures and pathways. J Affect Disord.

[14] Schlax J, Jünger C, Beutel ME, Münzel T, Pfeiffer N, Wild P, Blettner M, Kerahrodi JG, Wiltink J, Michal M (2019). Income and education predict elevated depressive symptoms in the general population: results from the Gutenberg health study. BMC Public Health.

[15] Kim JM, Shin IS, Yoon JS, Stewart R (2002). Prevalence and correlates of late-life depression compared between urban and rural populations in Korea. Int J Geriatr Psychiatry.

[16] Patel V, Burns JK, Dhingra M, Tarver L, Kohrt BA, Lund C (2018). Income inequality and depression: a systematic review and meta-analysis of the association and a scoping review of mechanisms. World Psychiatry.

[17] Kawachi I, Kennedy BP (1999). Income inequality and health: pathways and mechanisms. Health Serv Res.

[18] Kawachi I, Subramanian SV, Almeida-Filho N (2002). A glossary for health inequalities. J Epidemiol Community Health.

[19] Gero K, Kondo K, Kondo N, Shirai K, Kawachi I (2017). Associations of relative deprivation and income rank with depressive symptoms among older adults in Japan. Soc Sci Med.

[20] Murata C, Kondo K, Hirai H, Ichida Y, Ojima T (2008). Association between depression and socio-economic status among community-dwelling elderly in Japan: the Aichi Gerontological evaluation study (AGES). Health Place.

[21] Xue Y, Lu J, Zheng X, Zhang J, Lin H, Qin Z, Zhang C (2021). The relationship between socioeconomic status and depression among the older adults: the mediating role of health promoting lifestyle. J Affect Disord.

[22] Xu Y, Yang J, Gao J, Zhou Z, Zhang T, Ren J, Li Y, Qian Y, Lai S, Chen G (2016). Decomposing socioeconomic inequalities in depressive symptoms among the elderly in China. BMC Public Health.

[23] Niedhammer I, Coindre K, Memmi S, Bertrais S, Chastang JF (2020). Working conditions and depression in the French national working population: results from the SUMER study. J Psychiatr Res.

[24] Maruyama T (2017). Depressive symptoms and overwork among physicians employed at a university hospital in Japan. J Health Soc Sci.

[25] Nakamura K, Takachi R, Kitamura K, Saito T, Kobayashi R, Oshiki R, Watanabe Y, Kabasawa K, Takahashi A, Tsugane S, Iki M, Sasaki A, Yamazaki O (2018). The Murakami cohort study of vitamin D for the prevention of musculoskeletal and other age-related diseases: a study protocol. Environ Health Prev Med.

[26] Kabasawa K, Tanaka J, Nakamura K, Ito Y, Yoshida K, Takachi R, Sawada N, Tsugane S, Narita I (2020). Study design and baseline profiles of participants in the Uonuma CKD cohort study in Niigata, Japan. J Epidemiol.

[27] Radloff LS (1977). The CES-D scale: a self-report depression scale for research in the general population. Appl Psychol Meas.

[28] Kohout FJ, Berkman LF, Evans DA, Cornoni-Huntley J (1993). Two shorter forms of the CES-D depression symptoms index. J Aging Health.

[29] Yokoyama E, Kaneita Y, Saito Y, Uchiyama M, Matsuzaki Y, Tamaki T, Munezawa T, Oida T (2008). Cut-off point for the 11-item shorter form of the CES-D depression scale. Nihon Univ J Med.

[30] Chang-Quan H, Zheng-Rong W, Yong-Hong L, Yi-Zhou X, Qing-Xiu L (2010). Education and risk for late life depression: a meta-analysis of published literature. Int J Psychiatry Med.

[31] Winkleby MA, Jatulis DE, Frank E, Fortmann SP (1992). Socioeconomic status and health: how education, income, and occupation contribute to risk factors for cardiovascular disease. Am J Public Health.

[32] Geyer S, Hemström Ö, Peter R, Vågerö D (2006). Education, income, and occupational class cannot be used interchangeably in social epidemiology. Empirical evidence against a common practice. J Epidemiol Community Health.

[33] Cermakova P, Pikhart H, Kubinova R, Bobak M (2020). Education as inefficient resource against depressive symptoms in the Czech Republic: cross-sectional analysis of the HAPIEE study. Eur J Pub Health.

[34] Han KM, Han C, Shin C, Jee HJ, An H, Yoon HK, Ko YH, Kim SH (2018). Social capital, socioeconomic status, and depression in community-living elderly. J Psychiatr Res.

[35] Fang M, Mirutse G, Guo L, Ma X (2019). Role of socioeconomic status and housing conditions in geriatric depression in rural China: a cross-sectional study. BMJ Open.

[36] Herd P, Goesling B, House JS (2007). Socioeconomic position and health: the differential effects of education versus income on the onset versus progression of health problems. J Health Soc Behav.

[37] Cohen S, Janicki-Deverts D, Chen E, Matthews KA (2010). Childhood socioeconomic status and adult health. Ann N Y Acad Sci.

[38] Matthews KA, Flory JD, Muldoon MF, Manuck SB (2000). Does socioeconomic status relate to central serotonergic responsivity in healthy adults?. Psychosom Med.

[39] Swartz JR, Hariri AR, Williamson DE (2017). An epigenetic mechanism links socioeconomic status to changes in depression-related brain function in high-risk adolescents. Mol Psychiatry.

[40] Furihata R, Uchiyama M, Takahashi S, Konno C, Suzuki M, Osaki K, Kaneita Y, Ohida T (2011). Self-help behaviors for sleep and depression: a Japanese nationwide general population survey. J Affect Disord.

[41] Iwata N, Roberts CR, Kawakami N (1995). Japan-U.S. comparison of responses to depression scale items among adult workers. Psychiatry Res.

[42] Lappalainen PH (2019). Conflicts as triggers of personal growth: post-traumatic growth in the organizational setup. Sci Med J.

[43] An L, Liu C, Zhang N, Chen Z, Ren D, Yuan F, Yuan R, Bi Y, Ji L, Guo Z, Ma G, Xu F, Yang F, Zhu L, Robert G, Xu Y, He L, Bai B, Yu T, He G (2019). GRIK3 RS490647 is a common genetic variant between personality and subjective well-being in Chinese han population. Emerg Sci J.

[44] Ahmadi Sarbarzeh P, Karimi S, Jalilian M, Mosafer H (2020). Depression, anxiety, stress and social isolation in hepatitis patients. Sci Med J..

