# Supplementary material for: Education, household income, and depressive symptoms in middle-aged and older Japanese adults
Source: BMC Public Health. 2021 Nov 18;21:2120. doi: 10.1186/s12889-021-12168-8 (PMC8600755; doi:10.1186/s12889-021-12168-8)
Supplement: Supplementary file 1 — Additional file 1. [file 12889_2021_12168_MOESM1_ESM.docx]

Additional Table 1. Distribution of characteristics of all participants by sex (number with percentage)

Men Women Total

N=19504 N=18995 N=38499

Age (years)

<50 4165 (21.4%) 4479 (23.6%) 8644 (22.5%)

50-59 5776 (29.6%) 6029 (31.7%) 11805 (30.7%)

60-69 7292 (37.4%) 6513 (34.3%) 13805 (35.9%)

≥70 2271 (11.6%) 1974 (10.4%) 4245 (11.0%)

Marital status

Married 15649 (80.7%) 15416 (81.6%) 31065 (81.2%)

Never married 2216 (11.4%) 733 (3.9%) 2949 (7.7%)

Divorced, separated or bereaved 1528 (7.9%) 2741 (14.5%) 4269 (11.2%)

Education level

Junior high school 4858 (24.9%) 4620 (24.3%) 9478 (24.6%)

High school 9881 (50.7%) 9217 (48.5%) 19098 (49.6%)

Junior college 2454 (12.6%) 4405 (23.2%) 6859 (17.8%)

University or higher 2311 (11.9%) 753 (4.0%) 3064 (8.0%)

Household income (Yen/yr)

0-2,990,000 5913 (30.3%) 7073 (37.2%) 12986 (33.7%)

3,000,000-5,990,000 8659 (44.4%) 7316 (38.5%) 15975 (41.5%)

6,000,000-8,990,000 3274 (16.8%) 2991 (15.8%) 6265 (16.3%)

≥9,000,000 1658 (8.5%) 1615 (8.5%) 3273 (8.5%)

Occupation^1^

Sales and service 3013 (15.6%) 3853 (20.4%) 6866 (18.0%)

Office work 857 (4.4%) 2276 (12.0%) 3133 (8.2%)

Professional 5894 (30.5%) 2806 (14.8%) 8700 (22.8%)

Manual 5766 (29.8%) 2695 (14.3%) 8461 (22.1%)

No job or others 3793 (19.6%) 7278 (38.5%) 11071 (29.0%)

Area

Murakami 5726 (29.4%) 5764 (30.3%) 11490 (29.8%)

Uonuma 13778 (70.6%) 13231 (69.7%) 27009 (70.2%)

Disease history of

Cancer 1245 (6.4%) 1238 (6.5%) 2483 (6.5%)

Myocardial infarction 208 (1.1%) 22 (0.1%) 230 (0.6%)

Stroke 522 (2.7%) 245 (1.3%) 767 (2.0%)

Diabetes 1817 (9.3%) 923 (4.9%) 2740 (7.1%)

Activities of daily living

No disability 18408 (94.4%) 17909 (94.3%) 36317 (94.3%)

Having some disability^4^ 1009 (5.2%) 1013 (5.3%) 2022 (5.3%)

Physically dependent^5^ 87 (0.5%) 73 (0.4%) 160 (0.4%)

Depressive symptoms

CES-D scores ≥7 5800 (29.7%) 6423 (33.8%) 12223 (31.8%)

^1^Professional includes professional and management; manual includes security, farming/forestry/fishery, transportation, and labour services

^2^Trend of married persons

^3^Trend of manual workers

^4^Having some disability, but can go out independently

^5^Having disability, and cannot go out without assistance
